# Supplementary material for: Computational memory capacity predicts aging and cognitive decline
Source: Nat Commun. 2025 Mar 20;16:2748. doi: 10.1038/s41467-025-57995-0 (PMC11926346; doi:10.1038/s41467-025-57995-0)
Supplement: Supplementary file 1 — Supplementary Information [file 41467_2025_57995_MOESM1_ESM.pdf]

Supplementary Information

Computational memory capacity predicts aging and cognitive decline

Mite Mijalkov*, Ludvig Storm, Blanca Zufiria-Gerboles, Daniel Vereb, Zhilei Xu, Anna Canal-Garcia, Jiawei Sun, Yu-Wei Chang, Hang Zhao, Emiliano Gomez Ruiz, Massimiliano Passaretti, Sara Garcia-Ptacek, Miia Kivipelto, Per Svenningsson, Henrik Zetterberg, Heidi Jacobs, Kathy Lüdge, Daniel Brunner, Bernhard Mehlig, Giovanni Volpe*, Joana B. Pereira*

* Corresponding authors:

Mite Mijalkov (mite.mijalkov@ki.se) // Giovanni Volpe (giovanni.volpe@physics.gu.se) // Joana B. Pereira (joana.pereira@ki.se)

Supplementary Methods 1: Comparison between regional memory capacity and memory capacity derived from lesioning studies

Using a lesioning method, the memory capacity of a specific brain region can be quantified by measuring the difference in the global memory capacity before and after removing that region and all its connections from the reservoir network. Following the approach in the main manuscript, we calculated this "lesional memory capacity" for all regions across the high-density range (from 21% to 30%) and evaluated the area under the curve (AUC) in this range. To ensure consistency in the comparison, we used the same input signal and input weights for the regional memory and lesional memory capacity calculations of all brain regions.

In order to account for age-related effects, we evaluated the association between the lesional and regional memory capacity in a reference group of 125 young participants aged under 36 years[97] from the Cam-CAN cohort. Our findings show that the average memory capacities of all regions are strongly correlated with each other (Supplementary Fig. 1A, r = 0.84). Furthermore, this strong correlation remained high when we evaluated it for each participant, instead of averaging across the group (Supplementary Fig. 1B, r = 0.73 – 0.87, with a median value of 0.83).

These findings demonstrate that the method for calculating regional memory capacity as described in the main manuscript correlates well with the regional memory capacity derived from lesion studies.

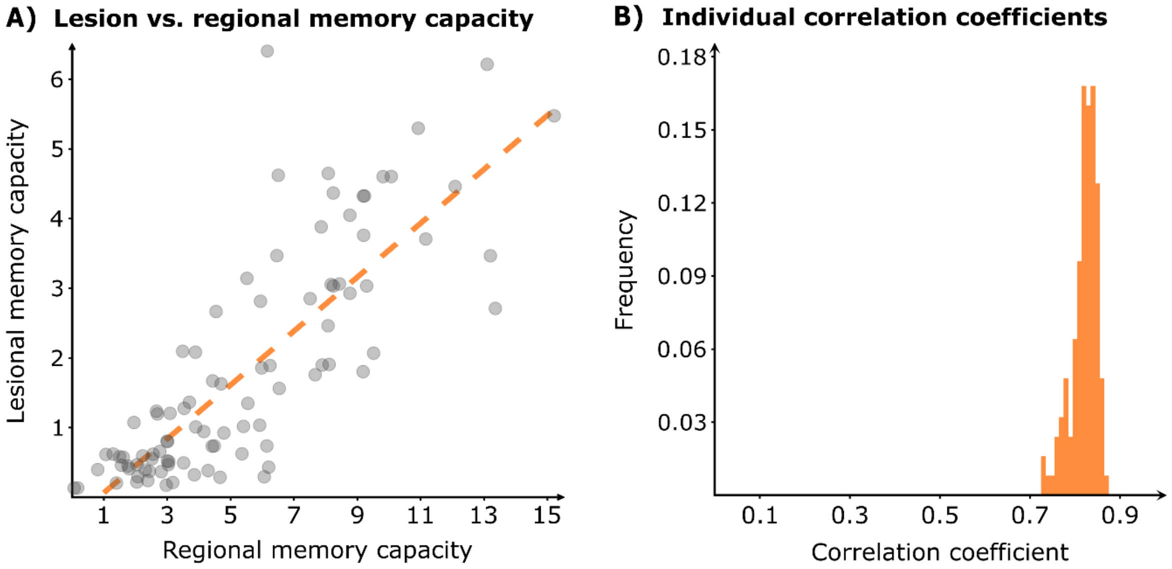

Supplementary Figure 1: Relationship between regional memory capacity and memory capacity derived from the lesioning method. A) Lesional memory capacity plotted as a function of the regional memory capacity for all regions of the AAL atlas. Both memory capacity values were calculated as an average across a reference group (n = 125) of young individuals (< 36 years) from the Cam-CAN cohort. The orange line represents the best line of fit between the two variables. **B)** Histogram of the correlations between lesional and regional memory capacity for all 125 subjects in the reference group.

Supplementary Methods 2: Small delays ($\tau = 1$ to $\tau = 5$) memory capacity as a function of age

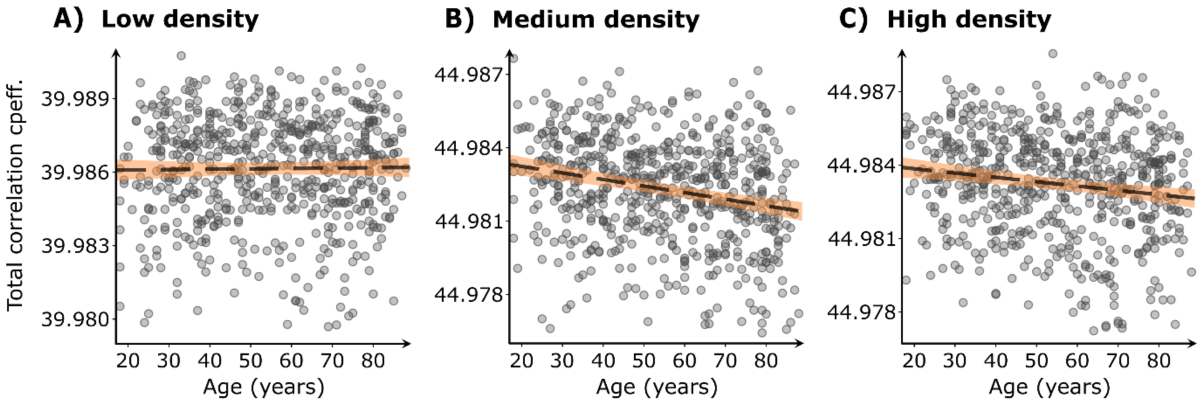

Supplementary Figure 2: The total Pearson's correlation coefficient between the signal predicted by the reservoir and the delayed input signal, calculated as the sum of the coefficients obtained at delays $\tau = 1$ to $\tau = 5$. Gray dots represent the AUC values of this coefficient when evaluated at **A)** low (2% - 10%), **B)** medium (11% - 20%), and **C)** high (21% to 30%) density (n = 636 subjects), dashed lines represent the best model fit and orange areas show the 95% CI for predictions. The AUC for each density values are calculated as outlined in the Methods, Statistical analysis section in the main text. Note the extremely small range of the y-axis, due to the fact that the network almost perfectly memorizes the input signals at small delays.

The results remained valid when we included these small delays in the calculation of the memory capacity. Specifically, we repeated the analyses shown in Fig. 1E – Fig. 1H in the main manuscript by evaluating the memory capacity using all delays between $\tau = 1$ to $\tau = 35$. Supplementary Fig. 3 shows that there was a significant decrease in the memory capacity of old individuals, where the differences increased as the density of the network increased. When evaluating the area under the curve for low (2%-10%), medium (11%-20%) and high (21%-30%) density ranges, we found that memory capacity could explain 4%, 6.9%, and 10.6% of the variance in age, respectively. These values were similar to the results presented in the manuscript, which were 3.98%, 6.87%, and 10.9% for the three density ranges, respectively.

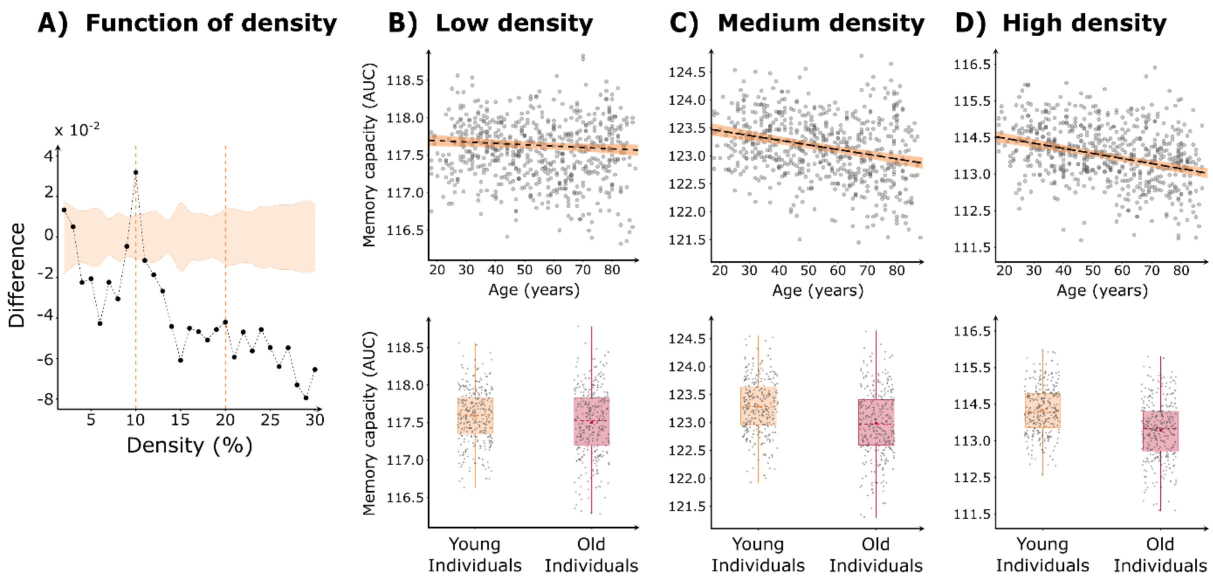

Supplementary Figure 3: Memory capacity in aging when memory capacity is defined for delays τ = 1 to τ = 35. A) Differences in global memory capacity between older (54-88 years old, n = 333) and younger (18-53 years old, n = 303) individuals. The plot shows the 95% confidence intervals (CI) in orange, while the circles represent the differences in global memory capacity as a function of network density; the significant differences fall outside the CI. Correlation between global memory capacity and age at **B)** low, **C)** medium, and **D)** high densities. In all cases, y-axis shows AUC values, circles indicate individual data points, dashed lines represent best model fit, and orange areas show 95% CI. Boxplots display AUC of the global memory capacity values for young (n = 303) and old (n = 333) subjects. They show the median (center line) and the mean (colored center circles) of each group, the box boundaries extend to the 25th and 75th percentiles of the sample with whiskers indicating non-outlier data.

Supplementary Methods 3: Differences in memory capacity between young and old individuals for extreme age groups

To assess how the differences between young and old individuals varied when comparing more extreme age groups, we defined the groups in the following way. Starting from a threshold of 53 years (as presented in the main manuscript), we progressively increased the threshold by 1 year for older subjects and decreased it by 1 year for younger ones, ensuring symmetry around the age of 53 years. The most extreme groups that we analyzed included individuals younger than 36 and older than 70. We then compared the means of the memory capacity at high-density range in young and old individuals and calculated Cohen's d to measure the effect size of these differences.

Supplementary Fig. 4A, 4B and 4C show the differences between the two groups as a function of density for three different thresholds. The differences follow the same pattern as Fig. 1E in the main manuscript. However, the magnitude of the largest difference increases from 0.08 for less extreme groups (Supplementary Fig. 4A), to 0.11 as the groups become more extreme (Supplementary Fig. 4B and 4C). This increasing magnitude of difference between old and young individuals can be also seen when plotting the AUC of the high-density range as a function of the age gap between the two groups (Supplementary Fig. 4D, $p_{max} < 10^{-4}$).

Finally, we calculated the Cohen’s d values for each comparison in Supplementary Fig. 4D, which ranged between 0.81 and 0.85, with a median value of 0.83 (Supplementary Fig. 4E). These analyses show that, although the mean differences are seemingly small in magnitude, they have large effect sizes (Cohen’s d > 0.8)[98], supporting the hypothesis that memory capacity is a good age-related biomarker.

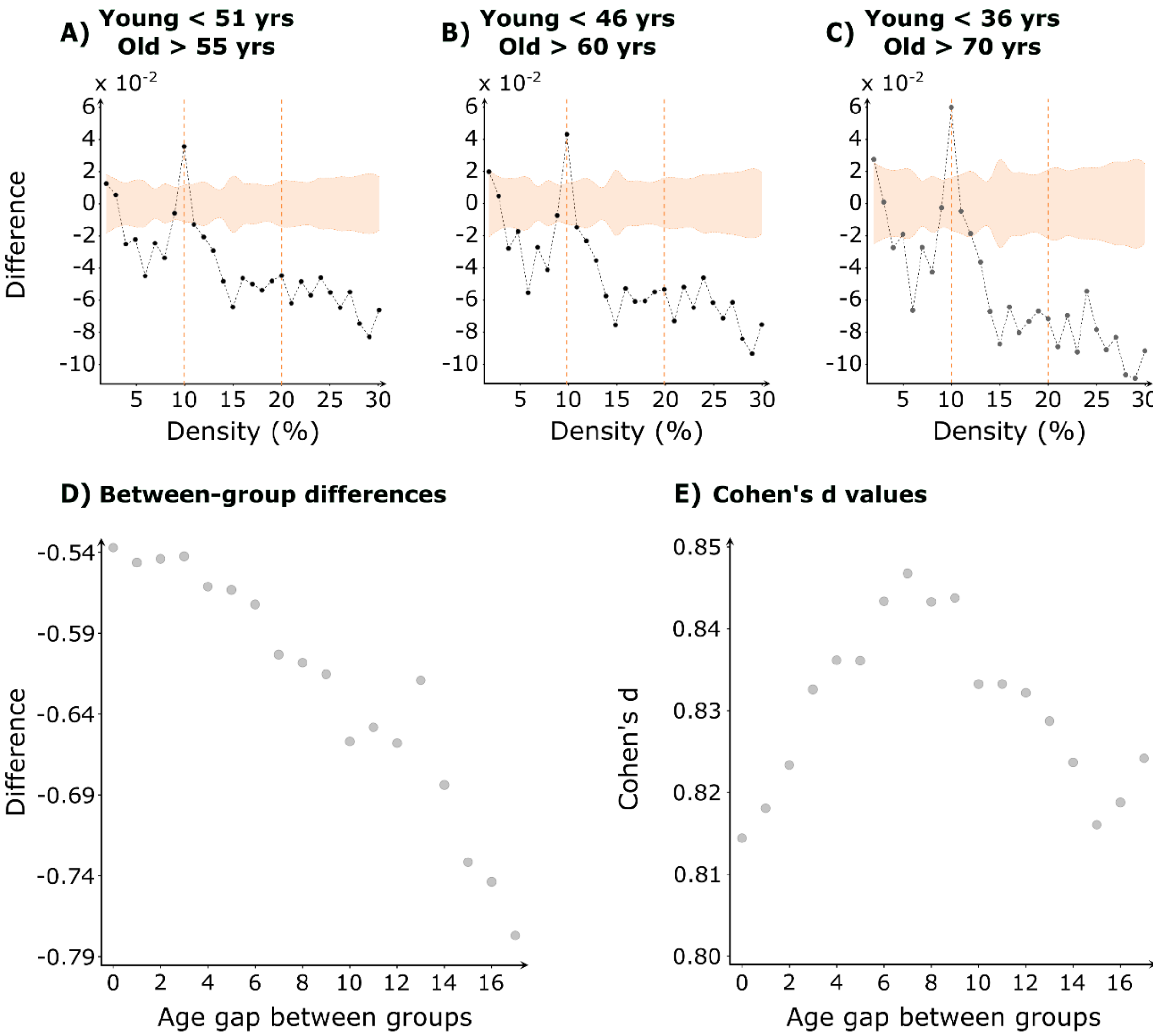

Supplementary Figure 4: Memory capacity in more extreme groups. Differences in memory capacity between old and young individuals as a function of network density for different thresholds to define old (young) groups **A)** 55, n = 313 (51, n = 284), **B)** 60, n = 264 (46, n = 230), and **C)** 70, n = 161 (36, n = 134). **D)** Differences in high-density memory capacity (calculated as old minus young) as a function of the age gap between old and young individuals. The age gap was centered around the age of 53 years. **E)** Cohen's-d value as a function of the age-gap between young and old individuals.

Supplementary Methods 4: Differences in memory capacity as a function of age threshold

To assess whether our results remained robust against variations of the age threshold of 53 years, we repeated our analysis by dividing the cohort into young and old individuals using thresholds ranging from 39 to 70 years. This range was chosen in order to ensure that both groups comprised at least 25% of the subjects in the cohort. Our results demonstrate that old individuals had lower memory capacity compared to young ones across the complete range (Supplementary Fig. 5A and 5B), which was significant across the entire range of thresholds ($p_{max} < 10^{-4}$).

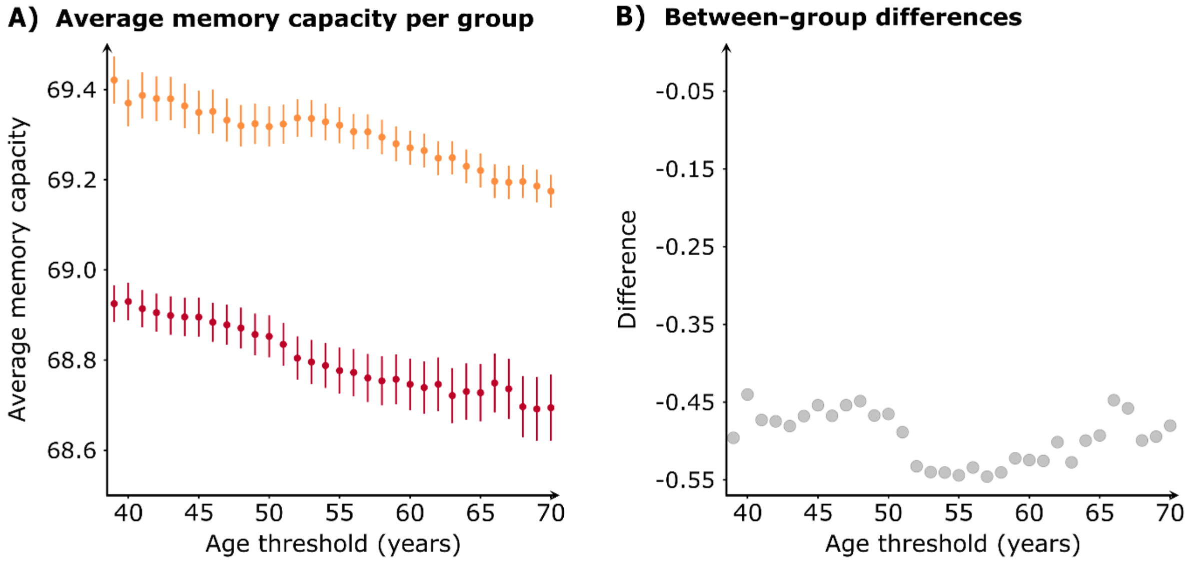

Supplementary Figure 5: Memory capacity as a function of the age threshold used to define young and old individuals. A) Average memory capacity for young (orange circles) and old (red circles) individuals as a function of the age-threshold used to categorize subjects into these groups. The error bars represent one standard error of the mean for each group. **B)** Between-group differences (calculated as old minus young) as a function of the age-threshold.

Supplementary Methods 5: Relationship between memory capacity and measures of network topology and brain structural integrity

Network topology was evaluated by the global efficiency and clustering coefficients, which are measures of network integration (the ability with which nodes communicate with each other through direct, efficiency connections) and network segregation (representing the clustered connectivity across all network nodes), respectively. Both measures were calculated on binary networks, using the BRAPH software[99].

Formally, global efficiency of a node is defined as the average of the inverse shortest path from that node to all other nodes in the network. The network global efficiency is calculated as the average of the global efficiencies of all network nodes:

$$GE = \frac{1}{N}\sum_{i \in N} GE_i = \frac{1}{N}\sum_{i \in N} \left(\frac{\sum_{j \in N, j \neq i} d_{ij}^{-1}}{N-1}\right),$$

where d_{ij} indicates the distance between nodes i and j (measured as the number of edges that need to be traversed to reach j starting from i) and N is the number of nodes.

Clustering coefficient of a network is calculated as the average of clustering coefficients for each node, which is defined as the number of closed triangles formed around each node:

$$C = \frac{1}{N}\sum_{i \in N} C_i = \frac{1}{N}\sum_{i \in N} \frac{2t_i}{d_i(d_i - 1)},$$

where t_i denotes the number of closed triangles and d_i is the degree (or number of connections) of node i.

For each network, we also calculated its average connectivity weight. It is important to note that the average weight was calculated after the normalization of the connectivity matrices to ensure their largest singular value is equal to unity, which was necessary in order to preserve the echo-state property of the reservoirs. Following the procedure in the main manuscript, we calculated global efficiency, clustering coefficient and average weight for each density in the high density range (21% to 30%). We then obtained the area under the curve (AUC) across this range and used these values for comparison to the high-density network memory capacity.

Supplementary Fig. 6 shows that memory capacity was associated with global efficiency ($r = 0.11$, $p = 0.005$, Supplementary Fig. 6A), average weight ($r = 0.37$, $p < 0.001$, Supplementary Fig. 6C) and mean FA values ($r = 0.32$, $p < 0.001$, Supplementary Fig. 6D). There was no significant association between the network memory capacity and network clustering coefficient (Supplementary Fig. 6B). This indicates that the changes in computational ability throughout aging are associated with the ability of brain regions to communicate directly with each other and the average strength of anatomical connectivity between them.

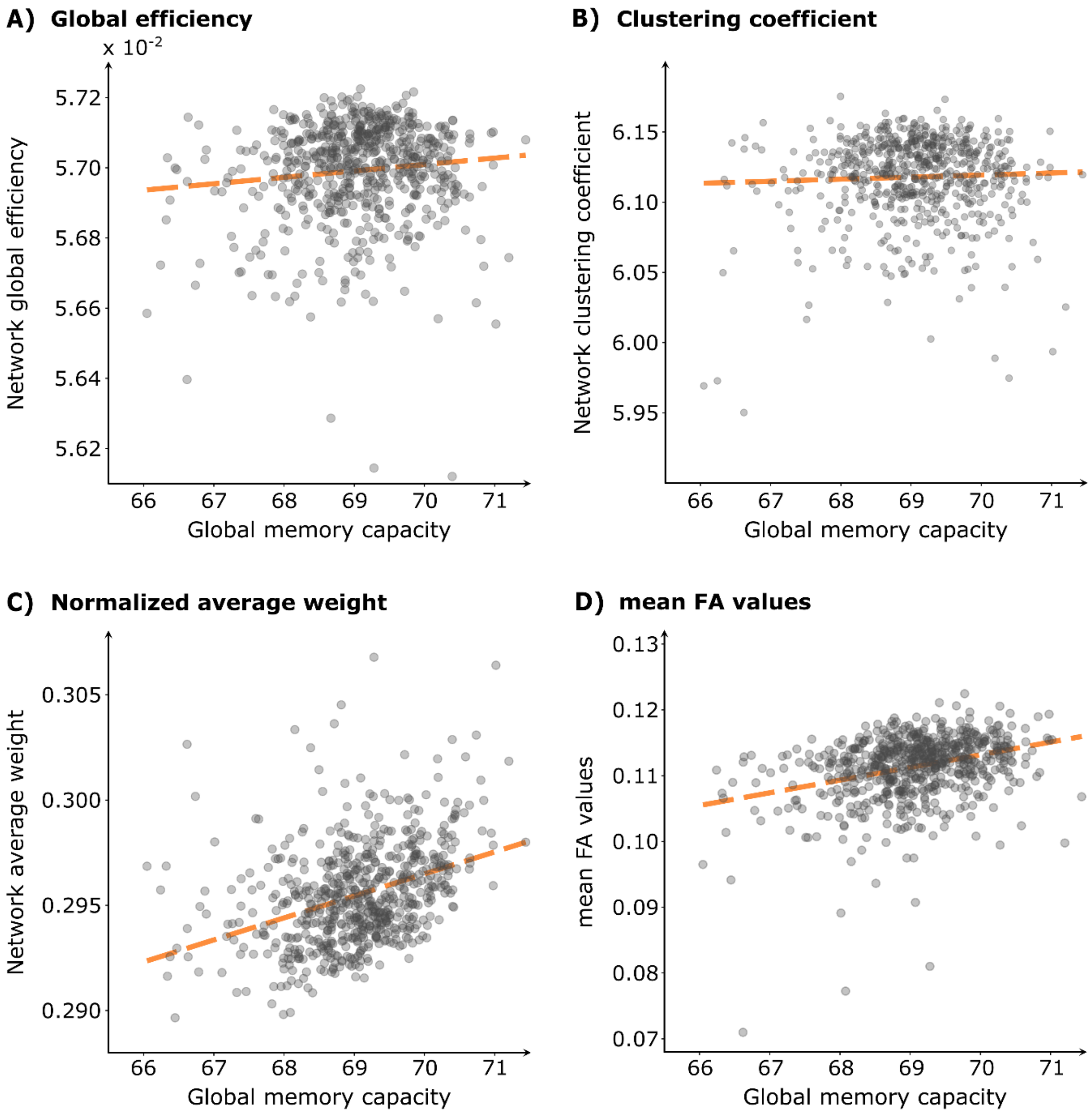

Supplementary Figure 6: Relationship between memory capacity, measures of network topology, and global mean FA values. Associations between global memory capacity and **A)** global efficiency, **B)** clustering coefficient, **C)** normalized average network weight and **D)** mean FA values. Each circle represents an individual subject of the Cam-CAN cohort; the correlations were evaluated across the entire cohort (n = 636). The orange lines represent the best line of fit between the two variables.

Supplementary Methods 6: Mediation analysis of the relationship between memory capacity and age

We conducted a mediation analysis which investigates whether the relationship between an independent variable (memory capacity) and a dependent variable (individual age) is mediated via a third variable (global efficiency, clustering coefficient, average network weight or mean FA). The mediation analysis was conducted using the "mediation" package[100] in R.

Clustering coefficient and global efficiency were not significant mediators of the relationship between memory capacity and age ($p = 0.56$ and $p = 0.19$). However, mean fractional anisotropy (FA) values and average weight were significant mediators, where the average normalized weight mediated only 8.8% ($p = 0.04$, Supplementary Fig. 7A) and mean FA values mediated 53.2% ($p < 0.001$, Supplementary Fig. 7B) of this relationship. These analyses demonstrate that the age-related differences in computational ability are partly mediated by the mean FA values and only weakly mediated by the average normalized network weight.

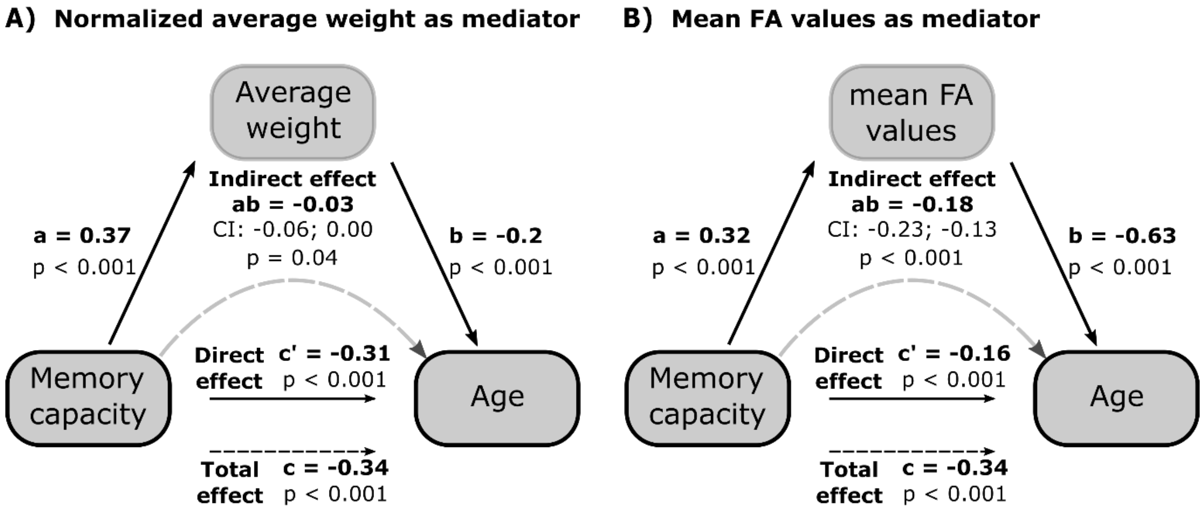

Supplementary Figure 7: Mediation analysis. Mediation analysis of the relationship between memory capacity and age with **A)** normalized average weight and **B)** mean FA values as mediators. Plots are showing effect estimates for the indirect (mediation), direct and total effect, where percentage of mediation was calculated as Indirect effect / Total effect * 100%.

Supplementary Methods 7: Relationship between age, memory capacity and FA values of individual tracts

We conducted a Partial Least Squares (PLS) regression analysis, where age was a dependent variable, while the independent variables included memory capacity, mean global FA, and the individual FA values in the following left (L) and right (R) tracts: Anterior thalamic radiation: ATR-L and ATR-R, Corticospinal tract: CST-L and CST-R, Cingulum (cingulate gyrus): CBC-L and CBC-R, Cingulum (hippocampus): CBH-L and CBH-R, Forceps major: FM-L and FM-R, Inferior fronto-occipital fasciculus: IFO-L and IFO-R, Inferior longitudinal fasciculus: ILF-L and ILF-R, Superior longitudinal fasciculus: SLF-L and SLF-R, Uncinate fasciculus: UF-L and UF-R, Superior longitudinal fasciculus (temporal part): SLFT-L and SLFT-R.

PLS performs a linear decomposition of the predictor and predicted variable matrix into latent variables (LVs), which were optimized so that the covariance between the resulting predictor and predicted matrix components (called factors and loadings, respectively) is maximal[101]. We used variable importance in the projection (VIP) scores in order to quantify how much each variable contributes to the prediction. The variables are calculated as the sum of PLS weights over LVs, weighted by the variance explained by each LV. Variables with VIP scores greater than 1 are defined as significant contributors to the prediction[102].

Supplementary Fig.8A shows that age was best explained by network memory capacity (VIP score = 2.02), followed by the FA values of the following tracts: Forceps major left (VIP score = 1.90) Inferior longitudinal fasciculus left (VIP score = 1.53), Cingulum (cingulate gyrus) right (VIP score = 1.36), Uncinate fasciculus right (VIP score = 1.30) and left (VIP score = 1.27), Inferior fronto-occipital fasciculus right (VIP score = 1.14), and Cingulum (cingulate gyrus) left (VIP score = 1.04). Altogether, they were able to explain 63.84% of the variance in age (Supplementary Fig. 8B).

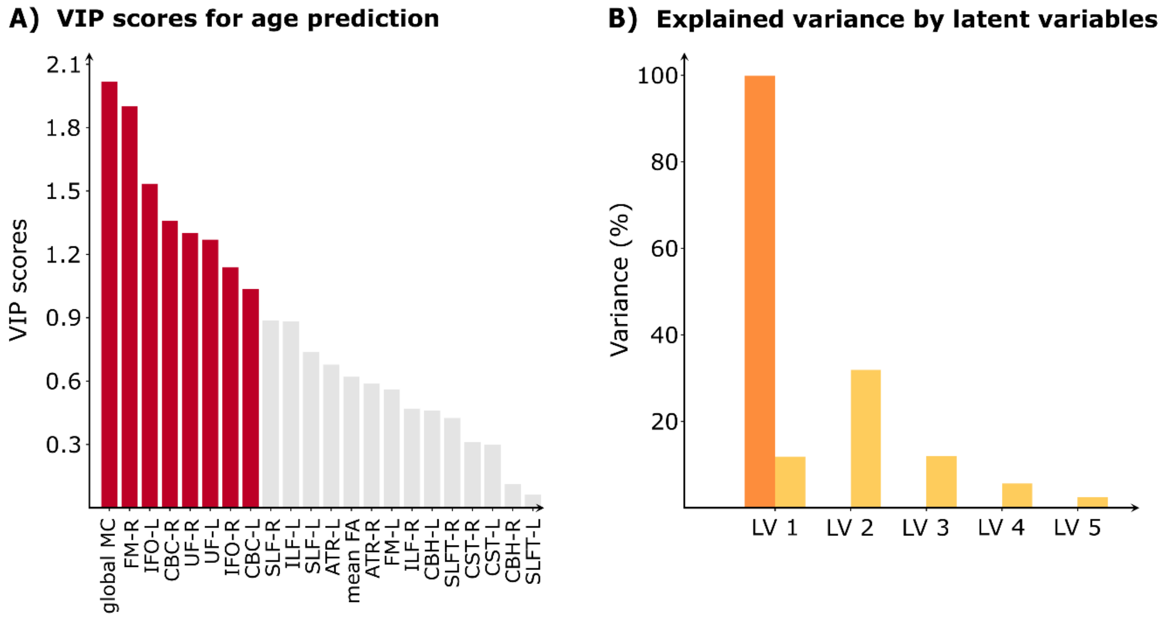

Supplementary Figure 8: Relationship between age, memory capacity, and FA values of different tracts. A) The VIP scores for all measures used as predictors, where the significant contributors are shown in red (VIP > 1) and non-significant contributors are shown in gray. **B)** Amount of variance for predicted and predictor variables (shown in orange and yellow,

respectively) explained by the corresponding latent variables. Abbreviations: ATR-L/ATR-R: Anterior thalamic radiation left/right, CST-L/CST-R: Corticospinal tract left/right, CBC-L/CBC-R: Cingulum (cingulate gyrus) left/right, CBH-L/CBH-R: Cingulum (hippocampus) left/right, FM-L/FM-R: Forceps major left/right, IFO-L/IFO-R :Inferior fronto-occipital fasciculus left/right, ILF-L/ILF-R: Inferior longitudinal fasciculus left/right, SLF-L/SLF-R: Superior longitudinal fasciculus left/right, UF-L/UF-R: Uncinate fasciculus left/right, SLFT-L/SLFT-R: Superior longitudinal fasciculus (temporal part) left/right.

Supplementary Methods 8: Age prediction using deep-learning models of varying complexity

The model presented in the main manuscript, with a layer configuration of [256, 512, 512, 256], showed optimal performance in predicting age (Supplementary Table 1). However, high correlation between true and predicted age could also be achieved with simpler models, reaching an average correlation coefficient of 0.64 for a 1 neuron model.

Complexity of multilayer perceptron model	Correlation between true and predicted age
[1]	0.64 ± 0.07
[4, 8, 8, 4]	0.63 ± 0.12
[8, 16, 16, 8]	0.67 ± 0.06
[16, 32, 32, 16]	0.69 ± 0.05
[32, 64, 64, 32]	0.69 ± 0.05
[64, 128, 128, 64]	0.72 ± 0.04
[128, 256, 256, 128]	0.74 ± 0.04
[256, 512, 512, 256]	0.74 ± 0.05
[512, 1024, 1024, 512]	0.73 ± 0.04
[1024, 2048, 2048, 1024]	0.71 ± 0.04

Supplementary Table 1: Performance of alternative deep-learning models with varying complexity for individual age prediction. Correlation between true and predicted age as a function of different deep-learning multilayer perceptron models (ranging from simpler to more complex from top to bottom). The average and the standard deviation values were derived by 10-fold cross-validation procedure.

Supplementary Methods 9: Locus coeruleus intensity vs. regional memory capacity

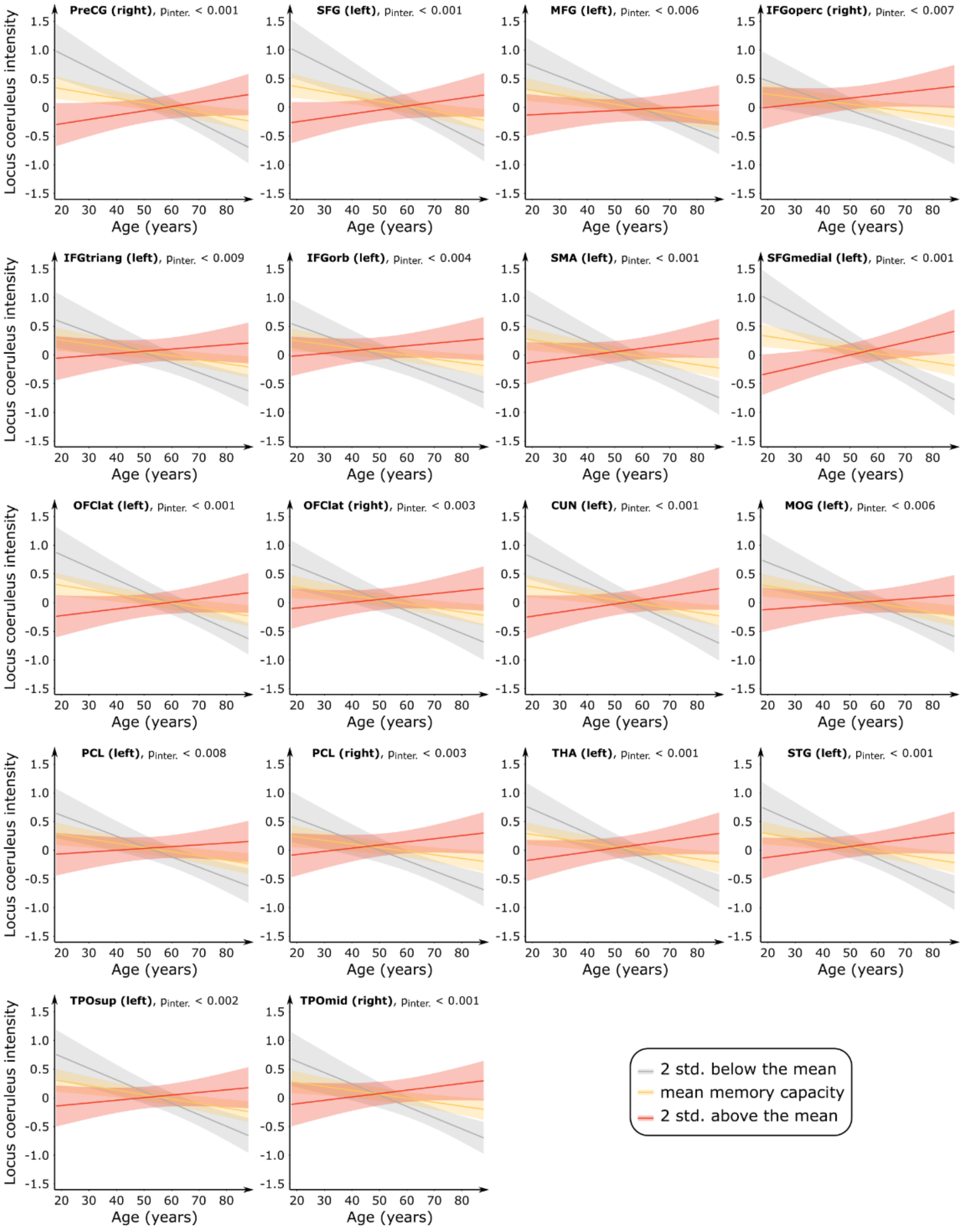

Supplementary Figure 9: Locus coeruleus intensity as a function of age, for different levels of memory capacity. Plots are shown for level 1: mean memory capacity, yellow; level 2: 2 standard deviations above the mean, gray; level 3: 2 standard deviations below the mean, red. The plotted values are the z-scores of the corresponding raw locus coeruleus intensity values. Interaction plots are shown only for regions that had significant interaction between regional memory capacity and age after correcting for multiple comparisons. The full list of the regions is as follows: precentral gyrus, superior frontal gyrus, middle frontal gyrus, inferior frontal gyrus (opercular part), inferior frontal gyrus (triangular part), inferior frontal gyrus (pars orbitalis), supplementary motor area, superior frontal gyrus (medial), left and right lateral orbital gyrus, cuneus, middle occipital gyrus, left and right paracentral lobule, thalamus, superior temporal gyrus, temporal pole (superior temporal gyrus), and temporal pole (middle temporal gyrus).

Supplementary Methods 10: Memory capacity and cognition

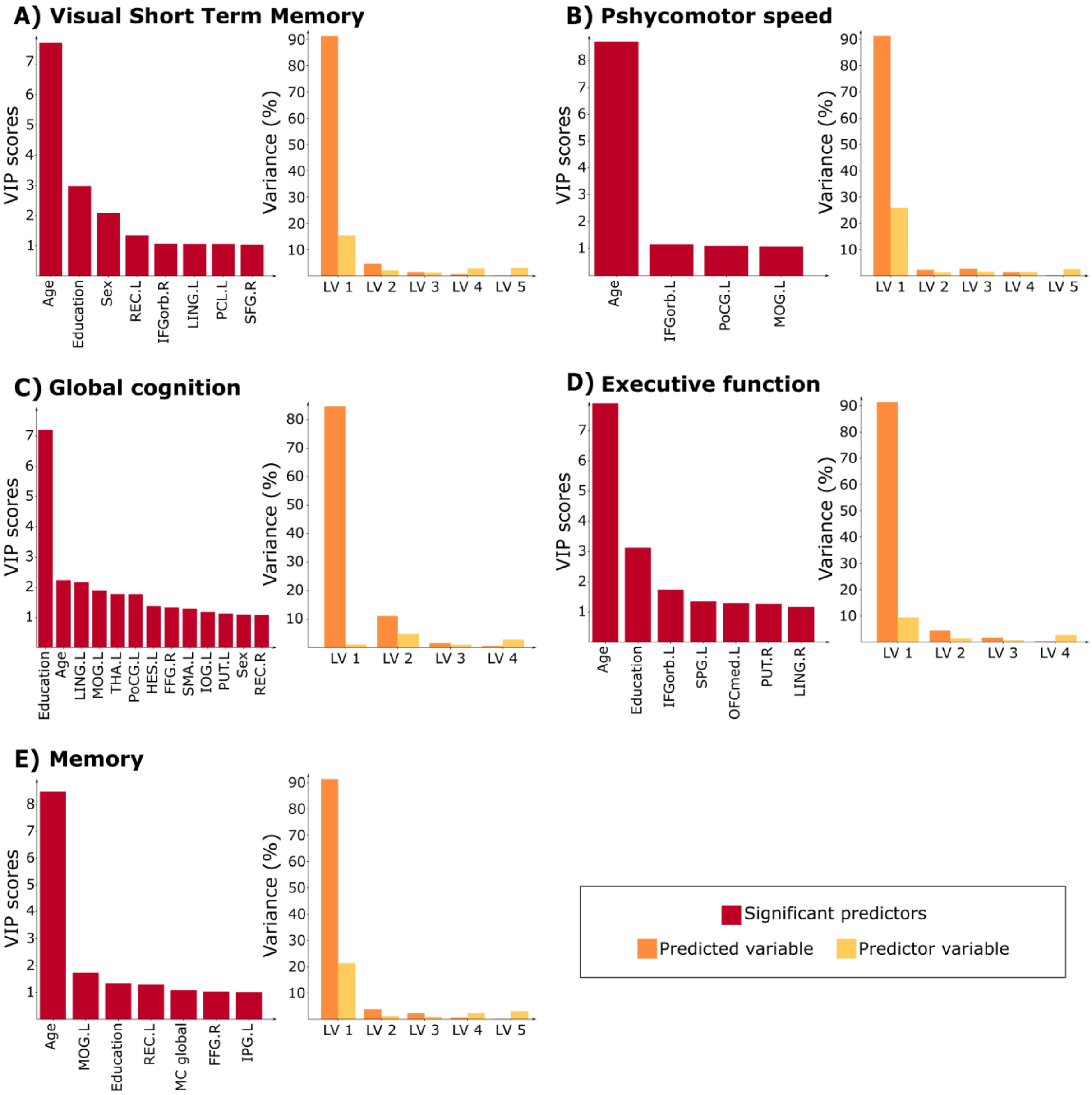

Supplementary Figure 10: Memory capacity and cognitive performance. Variable importance in projection (VIP) scores for all significant predictors and the amount of variance for predicted and predictor variables (shown in orange and yellow, respectively) explained by the corresponding latent variables. The partial least squares analysis was performed for individual test scores for the following domains: **A)** visual short term memory, **B)** psychomotor speed, **C)** global cognition, **D)** executive function, and **E)** memory. The predictors correspond to the ones outlined in the Results' Section, "Higher global and regional memory capacities are associated with better cognition".

Supplementary Methods 11: Group differences in global memory capacity in the LEMON cohort

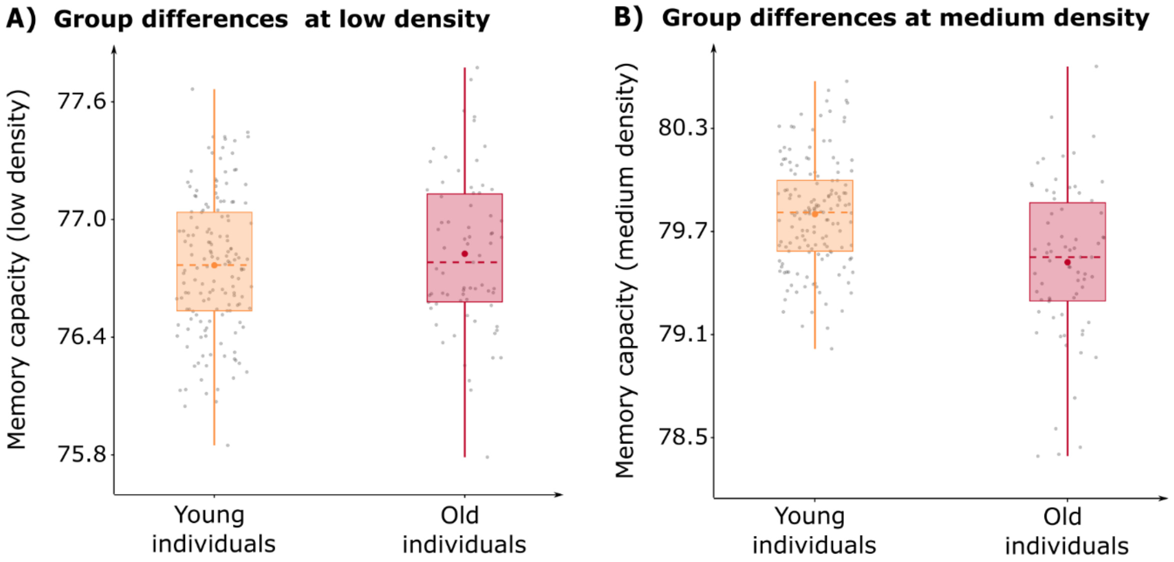

Supplementary Figure 11: Between-group differences in LEMON cohort. Boxplots showing network memory capacity values for young (n = 154) and old (n = 72) individuals, calculated at **A)** low and **B)** medium densities in the LEMON cohort. The boxplots show the median (center line) and the mean (colored center circles) of each group, the box boundaries correspond to the 25th and 75th percentiles of the sample with whiskers indicating non-outlier data.

Supplementary Methods 12: Normalization procedure for the whole-brain anatomical networks

We performed the normalization by setting the largest singular value of the reservoir networks, denoted by s_{SVD}, was equal to unity. This normalization was necessary in order to ensure that the reservoirs had the echo-state property, which allows the reservoir dynamics to synchronize with the input signal[103,104]. Therefore, the normalization was performed for each reservoir network (for each participant and for each density) independently of each other.

The choice to normalize s_{SVD} of each network to unity was guided by a recent paper[104] that outlined a procedure of choosing the network parameters in order to achieve the echo-state property. As demonstrated in this study[104], when "tanh" is used as an activation function for the reservoir nodes, normalizing the largest singular value of the reservoir's connectivity matrix to unity is a sufficient condition that guarantees the presence of the echo-state property throughout the simulation.

We performed additional analyses to assess whether our analyses remained robust against variations in this normalization procedure. Specifically, we performed the normalization by scaling the connectivity matrices (for each participant and at each density) such that their largest singular value, s_{SVD}, was in the range 0.1 to 1.5, where the range between 0.9 and 1 was further subdivided based on earlier studies showing that it could affect memory length of the network[105]. Supplementary Table 2 shows the global memory capacity obtained for these values of the normalization procedure across the entire cohort. Our findings show that highest global memory capacity can be obtained at $s_{SVD} = 0.96$. Nevertheless, both $s_{SVD} = 0.96$ and $s_{SVD} = 1$, provided similar prediction ability of age, with average correlation between predicted and true age across 10-fold cross-validation being 0.72 $\pm$ 0.04 and 0.72 $\pm$ 0.03, respectively.

Maximum singular value (s_{SVD})	Global memory capacity (high-density)	Maximum singular value (s_{SVD})	Global memory capacity (high-density)
0.1	0.77 - 0.98(0.87)	0.94	70.45 - 73.84(72.22)
0.2	4.09 - 5.08(4.56)	0.95	71.13 - 74.39(72.74)
0.3	12.95 - 14.17(13.60)	0.96	71.30 - 74.35(72.86)
0.4	20.53 - 22.55(21.59)	0.97	70.76 - 74.13(72.43)
0.5	27.63 - 29.52(28.67)	0.98	69.90 - 72.87(71.30)
0.6	36.32 - 38.55(37.40)	0.99	67.82 - 70.95(69.34)
0.7	44.73 - 46.95(45.96)	1.0	63.83 - 68.37(65.74)
0.8	54.22 - 57.44(56.00)	1.1	47.42 - 51.54(49.08)
0.9	66.29 - 69.99(68.02)	1.2	39.84 - 42.61(40.78)
0.91	67.62 - 71.16(69.25)	1.3	33.92 - 36.25(34.75)
0.92	68.87 - 72.06(70.40)	1.4	29.34 - 31.27(30.05)
0.93	69.89 - 73.12(71.41)	1.5	26.28 - 27.88(26.93)

Supplementary Table 2: Optimization of normalization parameters for the reservoir connectivity matrix. We calculated the global memory capacity for the cases when the maximal singular value of each network, s_{SVD}, is normalized in the range 0.1 to 1.5. The table shows the minimum and maximum memory capacity (median in parenthesis) across all individuals in the Cam-CAN cohort (n = 636).

To account for the possibility that each individual connectome can reach optimal memory performance at different s_{SVD} values, we recalculated the memory capacity by normalizing each connectome by its optimal s_{SVD} value (denoted by s_{SVD}^{opt}). Our results indicate that the majority of the participants (n = 443, 69.7%) achieved optimal memory capacity at a value of 0.96, consistent with Supplementary Table 2. Furthermore, 2 participants (0.3%) showed optimal performance at $s_{SVD}^{opt} = 0.94$, 182 participants (28.6%) at $s_{SVD}^{opt} = 0.95$, and 9 participants (1.4%) at $s_{SVD}^{opt} = 0.97$.

We investigated the age-related differences in the optimal memory capacity and s_{SVD}^{opt} by dividing the cohort into young and old individuals using age thresholds from 39 to 70 years (Supplementary Methods 4). We observed no significant differences in s_{SVD}^{opt} between the age groups (p values ranging from 0.29 to 0.96), suggesting no relationship between age and the singular value that leads to optimal performance. However, when comparing the optimal memory capacity across these age thresholds, we found that old individuals consistently had lower memory capacity than young individuals (Supplementary Fig. 12). These results align

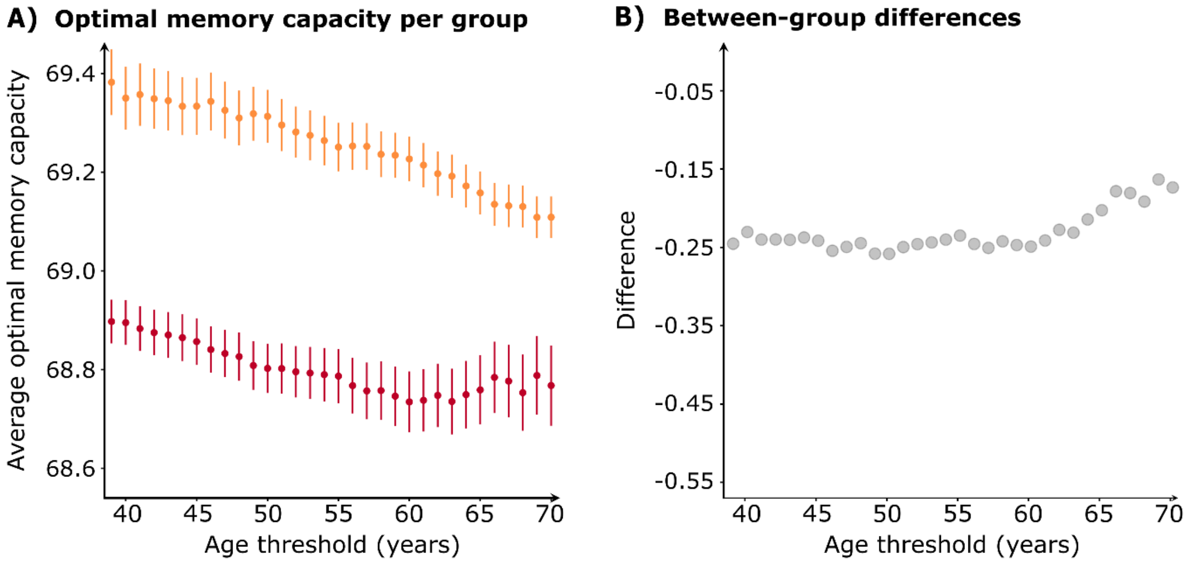

with those shown in Supplementary Fig. 5, indicating that memory capacity exhibits similar trends across different normalization procedures.

Supplementary Figure 12: Optimal memory capacity for each individual as a function of the age threshold used to define young and old individuals. A) Average optimal memory capacity for young (orange circles) and old (red circles) individuals as a function of the age-threshold used to categorize subjects into these groups. The error bars represent one standard error of the mean for each group. **B)** Between-group differences (calculated as old minus young) as a function of the age-threshold.

Supplementary Methods 13: Robustness against different preprocessing pipelines

To assess whether our analyses were robust against preprocessing pipelines, we used a probabilistic approach to derive the anatomical connectomes. Specifically, eddy-corrected and skull-stripped DWI images underwent fiber orientation estimation using a ball-and-stick model adjusted for multi-shell data with FSL bedpostX[106]. Similarly to the deterministic tractography analysis, the AAL2 atlas was used for parcellation to define seed and target masks for the tractography. Whole-brain connectomes were built for each subject by tracing a probabilistic distribution of streamlines from each region to all other regions using FSL probtrackx2[107]. Connectivity between two regions was defined as the number of streamlines connecting the two regions. After calculating the connectome of each individual, we repeated the analysis outlined in the main manuscript. Specifically, we binarized each connectome at the high-densities (21% - 30%) and calculated the global memory capacity for each density. Finally, we calculated the area under the curve (AUC) for the memory capacity across this range, and used these AUC values as dependent variables in a linear model, with age as an independent variable and sex as a covariate.

Supplementary Fig. 13A shows that memory capacity decreased with age, with age accounting for 18.8% of the variance in memory capacity ($p_{age} < 10^{-30}$). Our comparisons between groups of young and old (Supplementary Fig. 13B) further confirmed that older individuals (54-88 years old) exhibited significantly lower memory capacity compared to younger individuals (18-53 years old). These findings are in line with the findings presented in the main manuscript (Fig. 1H) and demonstrate that memory capacity is robust against different preprocessing pipelines.

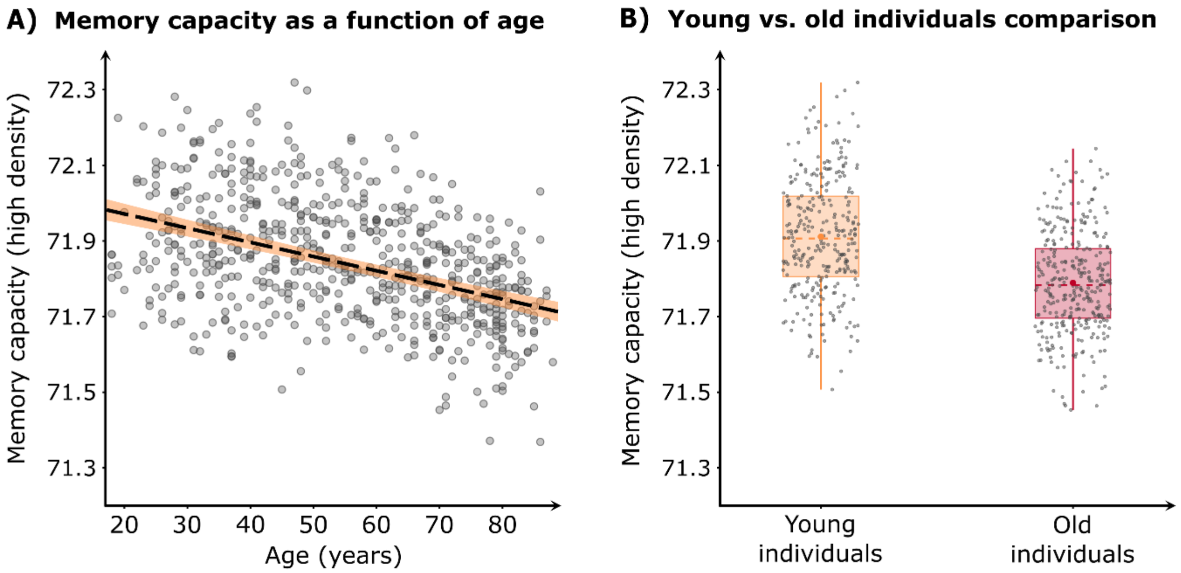

Supplementary Figure 13: Memory capacity for probabilistic tractography connectomes. A) Correlation between global memory capacity and age. The y-axis shows the AUC values of the memory capacity values calculated at the high-density range (21% to 30%). Orange area represents the 95% CI for predictions, the dashed lines indicate the best model fit, and the dots represent global memory capacity values for all individuals (n = 636). **B)** Differences in global memory capacity between older (54-88 years old, n = 333) and younger (18-53 years old, n = 303) individuals. Boxplots show the median (center line) and the mean (colored center circles)

of each group, and the box boundaries extend to the 25th and 75th percentiles of the sample with whiskers indicating non-outlier data.

Supplementary References

97. Van Essen, D. C. *et al.* The WU-Minn Human Connectome Project: An overview. *NeuroImage* **80**, 62–79 (2013).
98. Cohen, J. *Statistical Power Analysis for the Behavioral Sciences*. (Routledge, 2013).
99. Mijalkov, M. *et al.* BRAPH: A graph theory software for the analysis of brain connectivity. *PLOS ONE* **12**, e0178798 (2017).
100. Tingley, D., Yamamoto, T., Hirose, K., Keele, L. & Imai, K. mediation: R package for causal mediation analysis. *UCLA Stat. Stat. Assoc.* (2014).
101. Abdi, H. & Williams, L. J. Partial Least Squares Methods: Partial Least Squares Correlation and Partial Least Square Regression. in *Computational Toxicology: Volume II* (eds. Reisfeld, B. & Mayeno, A. N.) 549–579 (Humana Press, Totowa, NJ, 2013). doi:10.1007/978-1-62703-059-5_23.
102. Chong, I.-G. & Jun, C.-H. Performance of some variable selection methods when multicollinearity is present. *Chemom. Intell. Lab. Syst.* **78**, 103–112 (2005).
103. Lukoševičius, M. & Jaeger, H. Reservoir computing approaches to recurrent neural network training. *Comput. Sci. Rev.* **3**, 127–149 (2009).
104. Storm, L., Gustavsson, K. & Mehlig, B. Constraints on parameter choices for successful time-series prediction with echo-state networks. *Mach. Learn. Sci. Technol.* **3**, 045021 (2022).
105. Gallicchio, C., Micheli, A. & Pedrelli, L. Deep reservoir computing: A critical experimental analysis. *Neurocomputing* **268**, 87–99 (2017).
106. Jbabdi, S., Sotiropoulos, S. N., Savio, A. M., Graña, M. & Behrens, T. E. J. Model-based analysis of multishell diffusion MR data for tractography: How to get over fitting problems. *Magn. Reson. Med.* **68**, 1846–1855 (2012).
107. Behrens, T. E. J., Berg, H. J., Jbabdi, S., Rushworth, M. F. S. & Woolrich, M. W. Probabilistic diffusion tractography with multiple fibre orientations: What can we gain? *NeuroImage* **34**, 144–155 (2007).
