# Supplementary material for: Computational memory capacity predicts aging and cognitive decline
Source: Nat Commun. 2025 Mar 20;16:2748. doi: 10.1038/s41467-025-57995-0 (PMC11926346; doi:10.1038/s41467-025-57995-0)
Supplement: Supplementary file 2 — Reporting Summary [file 41467_2025_57995_MOESM2_ESM.pdf]

nature portfolio

Corresponding author(s): Mite Mijalkov, Giovanni Volpe, Joana B Pereira

Last updated by author(s): Jan 8, 2025

Reporting Summary

Nature Portfolio wishes to improve the reproducibility of the work that we publish. This form provides structure for consistency and transparency in reporting. For further information on Nature Portfolio policies, see our Editorial Policies and the Editorial Policy Checklist.

Statistics

For all statistical analyses, confirm that the following items are present in the figure legend, table legend, main text, or Methods section.

n/a	Confirmed	
☐	☒	The exact sample size (*n*) for each experimental group/condition, given as a discrete number and unit of measurement
☐	☒	A statement on whether measurements were taken from distinct samples or whether the same sample was measured repeatedly
☐	☒	The statistical test(s) used AND whether they are one- or two-sided<br>*Only common tests should be described solely by name; describe more complex techniques in the Methods section.*
☐	☒	A description of all covariates tested
☐	☒	A description of any assumptions or corrections, such as tests of normality and adjustment for multiple comparisons
☐	☒	A full description of the statistical parameters including central tendency (e.g. means) or other basic estimates (e.g. regression coefficient) AND variation (e.g. standard deviation) or associated estimates of uncertainty (e.g. confidence intervals)
☐	☒	For null hypothesis testing, the test statistic (e.g. *F*, *t*, *r*) with confidence intervals, effect sizes, degrees of freedom and *P* value noted<br>*Give P values as exact values whenever suitable.*
☐	☒	For Bayesian analysis, information on the choice of priors and Markov chain Monte Carlo settings
☐	☒	For hierarchical and complex designs, identification of the appropriate level for tests and full reporting of outcomes
☐	☒	Estimates of effect sizes (e.g. Cohen's *d*, Pearson's *r*), indicating how they were calculated

Our web collection on statistics for biologists contains articles on many of the points above.

Software and code

Policy information about availability of computer code

Data collection	No software was used for data collection.
Data analysis	The reservoir computing paradigm and memory capacity were calculated using a modified version of BRAPH 2.0 using the pipeline "Memory Capacity". BRAPH software can be freely downloaded from http://braph.org/ (https://github.com/braph-software/BRAPH-2), along with detailed user manuals of how to upload and analyze the data. The modified version of BRAPH 2.0 is hosted on https://github.com/braph-software/MemoryCapacity.<br><br>All deep learning methods were developed in Python (v3.8.10, https://www.python.org/) using the following open-source packages: Pandas (v1.5.3) and Numpy (v1.21.3) for data handling and Scikit-learn(v1.2.1) and TensorFlow (v2.7.0-gpu, Keras based) for neural network development and evaluation.<br><br>The diffusion-weighted scans were preprocessed using FSL (v6.0.7, https://fsl.fmrib.ox.ac.uk/fsl/fslwiki), and were corrected for motion and eddy current using EDDY and skull stripped using BET, both of which are distributed with FSL. The functional MRI scans were preprocessed using fMRIPrep (v20.2.4, https://fmriprep.org/en/stable/), where registration to a standard MNI152 template space was performed using Freesurfer (v7.4.1) and ANTs (v2.4.4). Magnetization transfer-weighted images were skull stripped using FSL BET and nonlinear registration as implemented in ANTs was applied to transform them to MNI space. The regression analysis using tract-based spatial statistics (TBSS) was performed in FSL. The group-average resting-state network maps were obtained using temporal concatenation ICA as implemented in FSL MELODIC (v3.15). The connectivity networks were calculated using a deterministic tractography pipeline implemented in DSI Studio (v2023.07.08, http://dsi-studio.labsolver.org).

For manuscripts utilizing custom algorithms or software that are central to the research but not yet described in published literature, software must be made available to editors and reviewers. We strongly encourage code deposition in a community repository (e.g. GitHub). See the Nature Portfolio guidelines for submitting code & software for further information.

Data

Policy information about availability of data

All manuscripts must include a data availability statement. This statement should provide the following information, where applicable:

- Accession codes, unique identifiers, or web links for publicly available datasets
- A description of any restrictions on data availability
- For clinical datasets or third party data, please ensure that the statement adheres to our policy

The data used in this study was obtained from Cambridge Centre for Ageing and Neuroscience (Cam-CAN) cohort (https://www.cam-can.org/). As a replication cohort, we used the Leipzig Mind-Brain-Body Dataset – LEMON, which can be accessed at http://fcon_1000.projects.nitrc.org/indi/retro/MPI_LEMON.html. Both cohorts are open-access and require an application for access. Source data are provided with this paper.

Research involving human participants, their data, or biological material

Policy information about studies with human participants or human data. See also policy information about sex, gender (identity/presentation), and sexual orientation and race, ethnicity and racism.

Reporting on sex and gender	In both cohorts, gender was recorded based on self-reporting by the participants. The Cam-CAN cohort comprises 636 individuals (315 male and 321 female), while the LEMON cohort includes 226 participants categorized into groups of young (154 total, 109 male, 45 female) and old (72 total, 36 male, 36 female) individuals.<br><br>The current manuscript presents general findings that do not apply to only one sex/gender. In the study design, the participants' self-reported gender was included as a covariate in all analyses (including linear modeling, partial least squares modeling, and statistical comparisons) in order to account for its effects on structural brain connectivity.
Reporting on race, ethnicity, or other socially relevant groupings	The manuscript does not subdivide the participants according to race/ethnicity or other socially relevant groupings, and does not study the possible effects of these variables on the results.
Population characteristics	Cam-CAN cohort: A lifespan cohort consisting of 636 individuals aged between 18 and 88 years (315 male and 321 female). The individuals were cognitively healthy, and free from any memory deficits, communication issues, or mobility difficulties. Additionally, participants did not have serious medical problems and have not been diagnosed with neurodegenerative diseases (including Alzheimer's Disease, Parkinson's Disease, Multiple Sclerosis), psychiatric conditions (such as bipolar disorder, schizophrenia), as well as head injuries or uncontrolled high blood pressure.<br><br>LEMON cohort: The cohort comprises 226 healthy participants divided into a young group (20 – 40 years, 109 male, 45 female), and an elderly group (55 – 80 years, 36 male, 36 female). Participants in both groups were cognitively healthy and had absence of cardiovascular diseases (for example, current and/or previous heart attack or congenital heart defect), psychiatric diseases, a history of neurological disorders, a history of malignant diseases, or a positive drug anamnesis.
Recruitment	Cam-CAN cohort: The cohort is a subset of a population-based, representative sample comprising approximately 3000 individuals aged 18 years and over. The sample was drawn from the general population using Primary Care Trust (PCT)'s lists within the Cambridge City (UK) area. Exclusions from this representative sample include term-time residents of colleges and universities, as well as participants deemed inappropriate for inclusion by their Primary Care Physician.<br><br>LEMON cohort: Participants in this cohort were recruited through public advertisements, leaflets, online advertisements, and information events at the University of Leipzig.
Ethics oversight	Both cohorts obtained written informed consent from all participants. The Cam-CAN cohort study was conducted in compliance with the Helsinki Declaration, and has been approved by Cambridgeshire 2 Research Ethics Committee (reference number: 10/H0308/50). The LEMON cohort study was also carried out in accordance with the Helsinki Declaration and the study protocol was approved by the ethics committee at the medical faculty of the University of Leipzig (reference number 154/13-ff). We did not use any additional data or re-contact the participants and signed data use agreements to analyze the data.

Note that full information on the approval of the study protocol must also be provided in the manuscript.

Field-specific reporting

Please select the one below that is the best fit for your research. If you are not sure, read the appropriate sections before making your selection.

☒ Life sciences ☐ Behavioural & social sciences ☐ Ecological, evolutionary & environmental sciences

For a reference copy of the document with all sections, see nature.com/documents/nr-reporting-summary-flat.pdf

Life sciences study design

All studies must disclose on these points even when the disclosure is negative.

Sample size	In the current manuscript, the sample size for both cohorts was determined by including all individuals with available demographic data who also had available diffusion-weighted imaging scans.<br><br>In the analysis of cognition, we considered a subsample in the cohort which consisted of individuals who had completed specific cognitive assessments relevant to the current manuscript. These assessments included the proverb comprehension test, face recognition for familiar faces test, visual short-term memory task, hotel task, and the "choice" response time task. Similarly, in the analysis involving structural integrity and functional activation, the sample included individuals with available structural and functional MRI scans, as well as magnetization transfer-weighted images.<br><br>Therefore, the determination of sample size was guided by the inclusion of all individuals who had available data for the relevant analysis, ensuring a comprehensive representation and the largest possible sample size for the analyses conducted.
Data exclusions	No data has been excluded from the analysis. All individuals from both cohorts that had available data for the relevant analyses have been included.
Replication	Both cohorts are based on a cross-sectional design i.e., data for each individual was measured at a single time point, with no repeated measurements.<br>The primary analysis in the current manuscript uses data from the Cam-CAN cohort, while the LEMON cohort serves as a replication cohort. We have obtained consistent findings in both cohorts, therefore, verifying the reproducibility of our results.
Randomization	The aim of the current manuscript is to propose an imaging marker for healthy aging, with participant age as the main variable for categorizing individuals into old and young groups. In the Cam-CAN cohort, we categorized individuals in groups of young (18-53 years old) and old (54-88 years old) individuals. In secondary analyses within this cohort (such as individual age prediction, analysis of structural integrity, and cognition), age was treated as a continuous variable, and individuals were not grouped.<br><br>In the LEMON cohort, age is considered a categorical variable, dividing the cohort into groups of young (20 – 40 years) and old (55 – 80 years) individuals. Therefore, we allocated participants to two groups (young and old individuals) based on the definitions provided by the cohort.
Blinding	The researchers were not blinded to the categorization of individuals into different groups. In the current manuscript, we obtain unbiased age prediction/classification by subdividing the dataset into train, validation, and test sets. We evaluate and report our findings on the test set, therefore ensuring that the model predictions are evaluated on previously unseen data.

Reporting for specific materials, systems and methods

We require information from authors about some types of materials, experimental systems and methods used in many studies. Here, indicate whether each material, system or method listed is relevant to your study. If you are not sure if a list item applies to your research, read the appropriate section before selecting a response.

Materials & experimental systems

n/a	Involved in the study
☒	☐ Antibodies
☒	☐ Eukaryotic cell lines
☒	☐ Palaeontology and archaeology
☒	☐ Animals and other organisms
☒	☐ Clinical data
☒	☐ Dual use research of concern
☒	☐ Plants

Methods

n/a	Involved in the study
☒	☐ ChIP-seq
☒	☐ Flow cytometry
☐	☒ MRI-based neuroimaging

Plants

Seed stocks	*Report on the source of all seed stocks or other plant material used. If applicable, state the seed stock centre and catalogue number. If plant specimens were collected from the field, describe the collection location, date and sampling procedures.*
Novel plant genotypes	*Describe the methods by which all novel plant genotypes were produced. This includes those generated by transgenic approaches, gene editing, chemical/radiation-based mutagenesis and hybridization. For transgenic lines, describe the transformation method, the number of independent lines analyzed and the generation upon which experiments were performed. For gene-edited lines, describe the editor used, the endogenous sequence targeted for editing, the targeting guide RNA sequence (if applicable) and how the editor was applied.*
Authentication	*Describe any authentication procedures for each seed stock used or novel genotype generated. Describe any experiments used to assess the effect of a mutation and, where applicable, how potential secondary effects (e.g. second site T-DNA insertions, mosiacism, off-target gene editing) were examined.*

Magnetic resonance imaging

Experimental design

Design type	Resting-state
Design specifications	n/a
Behavioral performance measures	n/a

Acquisition

Imaging type(s)	Diffusion-weighted imaging, resting-state functional MRI images, magnetization transfer ratio images
Field strength	3 Tesla
Sequence & imaging parameters	Cam-CAN cohort: The images were collected at a single site using a 3T Siemens TIM Trio scanner with a 32-channel head coil. The diffusion weighted data was acquired with a twice-refocused-spin-echo sequence, with 30 gradient directions for b-values 1000 and 2000s/mm2, while three images were acquired using a b-value of 0. The following parameters were used: TE=104 ms, TR=9100 ms, field of view (FOV)=192 mm×192 mm, voxel size=2mm×2mm×2mm, 66 axial slices, GRAPPA acceleration factor 2. The T1-weighted scans were acquired using a MPRAGE sequence with parameters: TR=2250 ms, TE=2.99 ms, FOV=256 mm×240 mm×192 mm, voxel size =1mm×1mm×1mm, GRAPPA factor 2. The resting-state functional MRI images were acquired with an echo planar imaging (EPI) sequence: TR=1970 ms, TE=30 ms, FOV=192 mm×192 mm, voxel size =3mm×3mm×4.44mm, 261 volumes, each containing 32 axial slices of thickness 3.7 mm. Magnetization Transfer Ratio (MTR) images were obtained from two MT-prepared Spoiled Gradient (SPGR) sequences: TR=30 ms (or TR=50 ms if SAR surpassed limits), TE=5 ms, FOV=192 mm×192 mm, voxel size =1.5mm×1.5mm×1.5mm, bandwidth =190 Hz/ px. The applied pulse was a Gaussian RF pulse with an offset frequency of 1950 Hz and duration 9984 µs (bandwidth=375 Hz, flip angle=500°).<br><br>LEMON cohort: The diffusion weighted data were acquired with multi-band accelerated sequence and an in-plane GRAPPA (acceleration factor 2). The parameters were as follows: 88 axial slices, voxel size =1.7mm×1.7mm×1.7mm, 60 gradient directions, b-value of 1000 s/mm2, TR=7000ms, TE=80ms, FA=90°, FOV=220mm, in addition to 7 images acquired using a b-value of 0.
Area of acquisition	Whole brain scan

Diffusion MRI ☒ Used ☐ Not used

Parameters: In the Cam-CAN cohort, the diffusion weighted data was acquired with a twice-refocused-spin-echo sequence, with 30 gradient directions for b-values 1000 and 2000s/mm2, while three images were acquired using a b-value of 0. The following parameters were used: TE=104 ms, TR=9100 ms, field of view (FOV)=192 mm×192 mm, voxel size=2mm×2mm×2mm, 66 axial slices, GRAPPA acceleration factor 2. In the LEMON cohort, the diffusion weighted data were acquired with multi-band accelerated sequence and an in-plane GRAPPA (acceleration factor 2). The parameters were as follows: 88 axial slices, voxel size =1.7mm×1.7mm×1.7mm, 60 gradient directions, b-value of 1000 s/mm2, TR=7000ms, TE=80ms, FA=90°, FOV=220mm, in addition to 7 images acquired using a b-value of 0.

Preprocessing

Preprocessing software	The DWI images from both cohorts were preprocessed simultaneously with the same pipeline in order to ensure consistency. The imaging data was preprocessed using FSL (v6.0.7, https://fsl.fmrib.ox.ac.uk/fsl/fslwiki). It was corrected for motion and eddy current using EDDY and skull stripped using BET, both of which are distributed with FSL, and then diffusion tensors were fit to the data using dtifit. Using the fitted tensors, we derived FA maps for each individual. In the case of probabilistic tractography, the eddy-corrected and skull-stripped DWI images underwent fiber orientation estimation using a ball-and-stick model adjusted for multi-shell data with FSL bedpostX. Whole-brain connectomes were built for each subject by tracing a probabilistic distribution of streamlines from each region to all other regions using FSL probtrackx2. Connectivity between two regions was defined as the number of streamlines connecting these two regions.<br><br>We preprocessed the functional scans from the Cam-CAN dataset using a standard pipeline in fMRIPrep (v20.2.4, https://fmriprep.org/en/stable/). We first removed the first two volumes to allow for steady state magnetization, followed by correcting the images for motion and slice timing effects. The functional images were then skull-stripped and, using a two-stage registration approach with Freesurfer (v7.4.1) and ANTs (v2.4.4), co-registered to a standard 2 mm resolution MNI152 template space. The resulting images additionally underwent motion correction using the Friston-24 head motion model and nuisance regression to remove confounding signals from the white matter and cerebrospinal fluid. The resulting volumes were spatially smoothed using an isotropic Gaussian kernel with 6 mm FWHM.<br><br>The magnetization transfer-weighted (MTW) images from the Cam-CAN cohort were first skull stripped using FSL BET. Then, we used bias field correction and nonlinear registration as implemented in ANTs to transform them to MNI space.
Normalization	Intensity normalization and co-registered to a standard 2 mm resolution MNI152 template space using a two-stage registration with Freesurfer and ANTs.

Normalization template

Standard 2 mm resolution MNI152

Noise and artifact removal

Regression of noise and motion-related artifacts using FSL FIX (https://fsl.fmrib.ox.ac.uk/fsl/fslwiki/FIX) and high-pass filtering with a cut-off of 2000s. Additionally, signals from the cerebrospinal fluid and white matter were removed via nuisance regression.

Volume censoring

We removed the first two volumes to allow for steady state magnetization.

Statistical modeling & inference

Model type and settings

Assessment of association between global memory capacity and white matter integrity: We conducted a regression analysis using tract-based spatial statistics (TBSS) as implemented in FSL. First, FA volumes of each participant were re-aligned to the FMRIB template in MNI space using nonlinear registration with FSL FNIRT. Then, a mean FA volume was derived by calculating the average of all participants' FA images. The mean FA volume was thresholded at FA=0.2 and skeletonized to represent the center of the white matter tracts. The template-aligned FA volumes of all participants were then projected to this mean FA skeleton. The relationship between global memory capacity and diffusion parameters was assessed using a general linear model (GLM) approach, with age and sex included as confound regressors in the model. The GLM was fitted voxel-wise and statistical inference was performed using a non-parametric permutation test as implemented in FSL randomise. The spatial relationship between voxels was taken into account by carrying out threshold-free cluster enhancement (TFCE) with 2D optimization.

Assessment of association between global memory capacity and voxel-wise functional connectivity within resting-state networks: We used a reference group of 125 participants under 36 years of age to derive a spatial template for well-known resting-state networks. Group-average resting-state network maps from the template cohort were obtained using temporal concatenation ICA as implemented in FSL MELODIC (v3.15). The number of components was determined automatically using the Laplacian approximation to the posterior distribution of the model order. Independent component spatial maps were thresholded by fitting a Gaussian-Gamma mixture-model to the Z-transformed intensity value histogram. Components were chosen for further analysis based on their spatiotemporal resemblance of known resting state networks. Individual versions of group-level resting-state network maps identified from the group ICA analysis were estimated using dual regression. The relationship between global memory capacity and functional connectivity was assessed voxel-wise for each RSN separately using a GLM-based non-parametric permutation approach similarly to the DTI analysis, with FSL randomize, controlling for age and biological sex. The analysis was constrained to the area under the thresholded group-average network maps.

Effect(s) tested

Define precise effect in terms of the task or stimulus conditions instead of psychological concepts and indicate whether ANOVA or factorial designs were used.

Specify type of analysis: ☒ Whole brain ☐ ROI-based ☐ Both

Statistic type for inference

Voxel-wise

(See Eklund et al. 2016)

Correction

Correction for multiple comparisons was performed by controlling the family wise error rate ($p < 0.05$).

Models & analysis

n/a	Involved in the study
☒	☐ Functional and/or effective connectivity
☒	☐ Graph analysis
☐	☒ Multivariate modeling or predictive analysis

Multivariate modeling and predictive analysis

We used area under the curve (AUC) analysis to summarize the global and regional memory capacity of each individual across low (2%-10%), medium (11%-20%), and high (21%-30%) network densities (the AUC was calculated by numerically integrating memory capacity values over the corresponding density range). The AUC values of the global memory capacity were included as dependent values in separate linear models, with age as independent values. All analysis used sex as a covariate.

We assessed the statistical significance of the differences between old and young individuals by nonparametric permutation tests with 10,000 permutations (considered significant for a two-tailed test of the null hypothesis at $p < 0.05$).

The relation between cognition and memory was evaluated by partial least squares analysis. We fitted a separate model for each cognitive test, which included age, sex, education, and global and regional memory capacity as predictors.

The deep-learning method used for the prediction of age was a multilayer perceptron. We used a neural network consisting of four hidden layers with 256, 512, 512, and 256 nodes. To prevent model overfitting, we applied dropout regularizations with a 10% rate between the hidden layers. The densely connected output layer was activated with a linear (for regression predictions) or sigmoid (for classification predictions) functions. The model performance was assessed using 10-fold cross validation. For each fold, the input data was divided into training, validation, and test datasets (80%, 10%, and 10%) and normalized (between 0 and 1). The multilayer perceptron was trained to minimize the mean absolute error (for the regression task or

age predictions in the Cam-CAN cohort) or binary cross entropy loss (for the binary classification task or the young versus old subjects classification in the LEMON cohort), using the Adam optimizer with a learning rate of 5 × 10-4 and batch size of 64 during 100 epochs. For each fold, we chose the final model with the lowest validation loss and evaluated its performance on the previously separated test data.
